# Methyl jasmonate fumigation enhances crop yield and delays physiochemical quality changes by modulating the secondary metabolism in green bell pepper

**DOI:** 10.1002/jsfa.70129

**Published:** 2025-08-20

**Authors:** Alicia Dobón‐Suárez, María J Giménez, Pedro J Zapata, María E García‐Pastor

**Affiliations:** ^1^ Department of Food Technology, Escuela Politécnica Superior de Orihuela Instituto de Investigación e Innovación Agroalimentario y Agroambiental (CIAGRO), University Miguel Hernández Alicante Spain; ^2^ Department of Applied Biology, Escuela Politécnica Superior de Orihuela Instituto de Investigación e Innovación Agroalimentario y Agroambiental (CIAGRO), University Miguel Hernández Alicante Spain

**Keywords:** *Capsicum annuum* L., antioxidant enzymes, phenolics, postharvest, quality losses

## Abstract

**BACKGROUND:**

The green bell pepper (*Capsicum annuum* L.), of the ‘Lamuyo’ type, is a highly valued vegetable owing to its excellent organoleptic and nutritional properties. However, these properties are subject to deterioration during postharvest storage, which in turn limits the shelf‐life of the pepper fruit. The present study examined the impact of preharvest treatment with 0.1 and 1.0 mmol L^−1^ methyl jasmonate (MeJA) administered via foliar spraying on crop yield and both physiochemical and functional fruit quality at harvest and throughout a 28‐day postharvest storage period at 7 °C.

**RESULTS:**

The study was conducted over two seasons (2020 and 2021) on green pepper fruit. The findings indicated that the application of MeJA had a favourable impact on crop yield in both growing seasons. Additionally, the treated peppers showed a 1.17‐fold reduction in weight loss and respiration rate, accompanied by augmented firmness, hue angle values, total soluble solids and total acidity in comparison to the controls. Applying 0.1 mmol L^−1^ MeJA was found to be the most effective way of maintaining quality at harvest and during postharvest storage over two consecutive seasons. Furthermore, MeJA treatments increased the levels of the antioxidant system by stimulating the activities of ascorbate peroxidase, catalase and peroxidase enzymes, as well as increasing the phenolic content and the total antioxidant activity. This could lead to an enhancement of the health benefits after the consumption of these treated peppers.

**CONCLUSION:**

The study demonstrates that the most efficacious concentration for enhancing yield and quality parameters by stimulating the secondary metabolism of peppers was 0.1 mmol L^−1^, a finding that was corroborated in the second season of 2021. The effects of MeJA on green pepper fruit may have significant commercial implications, as it has the potential to enhance quality at harvest and maintain it during postharvest storage. This may result in a delay in the onset of quality losses and the oxidative stress associated with fruit senescence, thereby extending the shelf‐life of the fruit after prolonged storage at optimal temperatures. © 2025 The Author(s). *Journal of the Science of Food and Agriculture* published by John Wiley & Sons Ltd on behalf of Society of Chemical Industry.

## INTRODUCTION

Bell peppers (*Capsicum annuum* L.), a member of the Solanaceae family, represent a significant agricultural and commercial crop, cultivated across tropical and subtropical regions globally.^1^, ^2^ The cultivated varieties of pepper fruit exhibit variation in fruit shape (block, conical, oblong), fruit size and colour.^3^ The most prevalent sweet pepper varieties are those with fleshy, block‐type fruit that is green (unripe) or red (ripe) in colour. The high level of consumer acceptance can be attributed to the functional compounds present in peppers, including polyphenols, flavonoids, vitamin C and carotenoids.^2^, ^4^ These parameters exhibit considerable variation in peppers depending on the physiological and maturity stage.^3^, ^5^, ^6^ Several factors, including the genetic environment (temperature, light, water and nutrient availability), production techniques used (including growth regulators, harvest date) and postharvest storage conditions,^7^, ^8^ have been identified as influencing these parameters. These metabolites may have beneficial effects on human health, and some may exert antioxidant activity, as they may be able to scavenge free radicals and oxygen.^9^ Conversely, a multitude of factors impact the quality of the pepper fruit, including fruit weight and firmness, as well as the soluble solids content.^10^ A strong positive correlation was observed between total soluble solids (TSS) and sugar content, which is frequently employed as an indicator of ripeness.^11^ Furthermore, the formation of non‐structural carbohydrates, which are utilised for the synthesis of phenolic compounds and antioxidants, has been associated with the sugar content of plants.^12^

In recent years, there has been a notable increase in the production of bell peppers. Nevertheless, postharvest losses of approximately 40% per annum are still recorded for this crop.^13^ Pepper is a highly perishable vegetable with a limited shelf‐life. Therefore, it is essential to ensure proper handling and adequate care for the maintenance of postharvest quality.^14^ The primary factors that negatively impact the postharvest quality of bell peppers during transportation, postharvest handling and storage at 7 °C (the optimal temperature to prevent chilling injury) are water loss and fruit softening.^1^, ^15^, ^16^ Consequently, bell peppers are vulnerable to a range of postharvest deterioration phenomena, including flaccidity, wilting, shrinkage, mechanical damage, fungal infections and deterioration.^17^ These factors collectively influence consumer acceptance of fruit. The accelerated ageing process observed in peppers is attributed to the generation of reactive oxygen species (ROS) and a reduction in antioxidant enzyme activity. These factors have a deleterious impact and are responsible for the induction of oxidative damage to cell components.^18^ Oxidative stress has been demonstrated to exacerbate enzymatic browning, which can ultimately result in a loss of shelf‐life. This is due to the peroxidizing of membrane lipids and the subsequent activation of softening‐related enzymes.^19^, ^20^ It is crucial to enhance the activities of redox enzymes, including catalase (CAT) and ascorbate peroxidase (APX), among others, in order to extend the shelf‐life of fruit and vegetable crops.^21^, ^22^, ^23^

A variety of postharvest technologies, including chemical and non‐chemical treatments, have been employed to preserve the quality of sweet peppers during storage.^13^, ^24^ However, the recent focus of postharvest research on peppers has been on the development of non‐polluting and non‐chemical treatments for the control of postharvest infections and the maintenance of metabolic processes in pepper fruits. In this regard, recent studies have documented a range of approaches for enhancing the quality and shelf‐life of pepper fruits through the utilisation of methodologies or materials, including organic manures, salicylic acid, cytokinin, chitosan, calcium and potassium thiosulfate, during the preharvest growth phase.^25^, ^26^, ^27^, ^28^, ^29^ Regarding elicitors, the action mechanism in the preservation of fruits and vegetables involves the activation of the plant's defence responses. This, in turn, results in the production of various defence molecules, including phytochemicals, antioxidants and enzymes, which help to protect the fruits and vegetables from microbial and oxidative degradation. Methyl jasmonate (MeJA) is a plant growth regulator derived from jasmonic acid (JA). It is involved in several biological processes and defence responses to biotic and abiotic stresses.^30^, ^31^ Previous studies have demonstrated that MeJA is a dose‐dependent signalling agent. For instance, García‐Pastor *et al*.^32^ found that high concentrations (5 and 10 mmol L^−1^) applied as preharvest treatments in table grapes delayed the ripening process and reduced the fruit weight. Conversely, lower concentrations (1, 0.1 and 0.01 mmol L^−1^) were observed to promote accelerated ripening.^32^ Furthermore, Otálora *et al*.^33^ demonstrated that the ameliorative impact of MeJA on heat stress was dependent on the specific variety of pepper plants, with the emergence of specific traits.^33^ This evidence suggests that the response of pepper cultivars to MeJA application differs.

On the other hand, recent findings indicate that the exogenous application of MeJA, either alone or in combination with low‐temperature conditioning, effectively alleviates the chilling injury of bell pepper.^1^, ^2^, ^34^ These treatments have resulted in the enhancement of cold resistance of green bell pepper fruit by regulating membrane lipid composition, glutathione metabolism and the antioxidant system, as revealed by multiomics insights. Moreover, Pu *et al*.^35^ have demonstrated that MeJA can mitigate the damage caused to pepper leaves by low temperature and low light, safeguarding the integrity of the cell membrane and enhancing the resilience of pepper seedlings in this environment.^35^ Notwithstanding these encouraging findings, there is currently no information available regarding the effects and preharvest application of MeJA for the purpose of enhancing the quality and shelf‐life of bell peppers under optimal stored conditions and at a non‐chilling injury temperature. Moreover, the precise mechanisms by which these beneficial effects are achieved remain unclear. This study aims to evaluate the impact of preharvest fumigation with MeJA on crop yield under greenhouse conditions, fruit quality and shelf‐life of bell peppers during 28‐day postharvest storage at 7 °C. Furthermore, it seeks to provide practical recommendations for growers to enhance the postharvest performance of this valuable crop. The central hypothesis was that treatment with MeJA would increase yield and delay changes in physiochemical quality (e.g., reduced weight loss and respiration, increased firmness), while also positively modulating the fruit's antioxidant system (both enzymatic and non‐enzymatic), thereby extending shelf‐life and mitigating oxidative stress.

## MATERIALS AND METHODS

### Plant material, experimental design and yield evaluation

The investigation was conducted in a commercial plot located in El Raal (Murcia, Spain), cultivated under greenhouse conditions with a plastic covering. The experimental procedure was carried out during both the 2020 and 2021 growing seasons. In January 2020, plants of the *Capsicum annuum* L. cultivar ‘Herminio’, designated ‘Lamuyo’, were planted. The duration of the experiment was 6 months, spanning the period from February to July in both 2020 and 2021, as evidenced in Table 1. Subsequently, 135 pepper plants were randomly selected and distributed across blocks to complete the experimental design. A total of 45 plants were included in each treatment, with three randomised blocks of 15 plants (*n* = 3). Treatments were administered at the onset of the morning in both consecutive seasons. The pepper plants were treated via foliar spray application with distilled water (pH 7.0) containing 1 mL L^−1^ Tween‐20, which served as the control group. In 2020, two concentrations of MeJA solutions, containing 1 mL L^−1^ Tween‐20, were employed: 0.1 and 1.0 mmol L^−1^ (reagent from Sigma‐Aldrich, Madrid, Spain; CAS Number: 39924‐52‐2). The optimal dose of MeJA (0.1 mmol L^−1^) was then replicated in 2021. The concentrations were selected based on previous research experiments conducted on other fruits, including sweet cherries, plums, table grapes, pomegranate and lemon fruit.^32^, ^36^, ^37^, ^38^, ^39^

**Table 1:** The number and date of application and harvest of treatments (control and MeJA at 0.1 and 1.0 mmol l^−1^) applied by foliar spraying to ‘Herminio’ green pepper plants throughout the 2020 and 2021 growing seasons

2020				2021			
Application number	Application date	Harvest number	Harvest date	Application number	Application date	Harvest number	Harvest date
A1	24 February	H1	06 April	A1	22 February	H1	07 April
A2	17 March	H2	20 April	A2	15 March	H2	22 April
A3	06 April	H3	04 May	A3	29 March	H3	04 May
A4	29 April	H4	14 May	A4	19 April	H4	14 May
A5	19 May	H5	26 May	A5	17 May	H5	27 May
A6	09 June	H6	04 June	A6	07 June	H6	03 June
A7	12 July	H7	16 June	A7	10 July	H7	11 June
		H8	26 June			H8	23 June
		H9	06 July			H9	05 July
		H10	27 July			H10	21 July

The letters ‘A’ and ‘H’ are used to denote the application and harvest, respectively.

A total of seven foliar spray applications were conducted throughout the crop cycle, as detailed in Table 1. The initial treatment was administered prior to the commencement of the flowering phase. The equidistance among application dates was approximately 21 days due to a staggered flowering cycle, except for the final application, which was conducted in proximity to the final commercial harvest. The application was selected based on the duration of the crop cycle of the specific pepper cultivar in question. The crop was cultivated in accordance with the established crop programme for the ‘Lamuyo’ pepper variety, which was overseen by the company. The plants were irrigated using drip systems with the requisite nutrient levels, in accordance with standard practice. The soil exhibited a pH value of 7.50 and a sandy loam texture. The green pepper fruits were harvested at the optimal phenological stage to ensure they were ready for commercial consumption, having reached their characteristic green colour and size.^5^ A total of ten harvest dates were conducted throughout the developmental and growth cycle of 2020 and 2021. These dates were selected in accordance with a staggered production schedule and the commercial criteria for harvesting green pepper fruit established by the company. The harvesting of the peppers commenced in April and continued until July in both the 2020 and 2021 seasons (Table 1). In both seasons, the period between the last MeJA administration and the final harvest date lasted 15 and 11 days, respectively (Table 1).

The mean daily temperatures for each month were recorded: April (14.58 °C), May (20.06 °C), June (23.33 °C) and July (25.98 °C) in 2020; and April (16.00 °C), May (19.30 °C), June (19.70 °C) and July (27.20 °C) in 2021, being recorded at a station situated in close proximity to the experimental greenhouses (38° 2′2.64 ″ north, 1° 1′ 18.9″ west). The relative humidity (RH) exhibited fluctuations between 66% and 89% throughout the course of the experiments. Crop yield was assessed at each harvest date throughout the crop cycle. The experimental design involved the establishment of blocks for each treatment in both growing seasons (2020 and 2021). The yield of green peppers was quantified until the conclusion of the final harvest. The accumulative yield was expressed in terms of kilograms per plant.

### Postharvest storage at optimal temperature

The green pepper fruits were subjected to analysis at harvest and following a 28‐day postharvest storage period. The experimental postharvest storage design was conducted for both green pepper seasons in 2020 and 2021. The former occurred on 4 May 2020, while the latter took place on 22 April 2021. The dates were selected in accordance with the optimal phenological stage.^5^ Green peppers, selected at random, without regard to any imperfections in appearance or internal quality, were extracted from each block per treatment and immediately transported to the laboratory. This procedure ensures the acquisition of a homogeneous sample from the different blocks, thereby ensuring the sample's representativeness of the commercial harvest. A total of 18 green peppers were selected for each treatment and sampling date (six peppers per replicate, with a total of three replicates; *n* = 3), after which they were weighed and stored at 7 °C and 85% RH. The green pepper fruits were subjected to analysis at harvest (day 0) and at 7, 14, 21 and 28 days of storage. Accordingly, 90 green pepper fruits were utilised for postharvest storage per treatment. The peppers were stored in uncovered, fruit‐safe boxes and distributed in three replicate batches (*n* = 3) of six peppers each. A total of 18 green peppers were selected for analysis, with each one representing a different treatment or sampling date. A series of measurements was taken for each sampling date, including weight loss, respiration rate, firmness, colour (hue angle), total soluble solids (TSS), total acidity (TA), total phenolic content (TPC), total antioxidant activity (TAA) and the enzymatic activity of the antioxidant enzymes.

### Weight loss, respiration rate and physiochemical traits

The weight loss (WL) of the treatments tested was determined by weighing each green pepper fruit individually at harvest (day 0) and at subsequent sampling dates (7, 14, 21 and 28 days) following storage. The WL was expressed as a percentage with respect to the initial weight of the green pepper fruit. The respiration rate (RR) was determined at room temperature by placing individual green pepper fruits in 2 L capacity glass jars, which were hermetically sealed for a period of 60 min. Subsequently, 1 mL of the atmosphere within the holder was withdrawn and employed for the quantification of carbon dioxide (CO₂) in a gas chromatograph (GC‐14B, Shimadzu, Kyoto, Japan) equipped with a thermal conductivity detector. The RR was then expressed as mg CO₂ kg^−1^ h^−1^.^26^ The firmness of each pepper fruit was assessed individually using a TX‐XT2i texturometer (Stable Microsystems, Godalming, UK). The texture analyser used a flat steel plate to determine the deformation of the equatorial fruit diameter by 5% due to force, at a speed of 100 mm min^−1^, expressed as a percentage of the initial measurement. The results were expressed as a force–deformation ratio (N mm^−1^). The colour of the equatorial region of the green pepper fruit was quantified at three points along the fruit's perimeter using a colorimeter (CFRC400, Minolta Camera Co., Tokyo, Japan). The results were expressed as the hue angle (hue°) parameter, with the CIELab coordinates employed for this purpose. The data regarding firmness and colour are the mean ± SE of three replicates (*n* = 3) of ten pepper fruits. Subsequently, one half of each of the six green peppers from each replicate was chopped and blended to create a uniform juice sample. The TSS were then determined in duplicate using a digital refractometer (PR‐101, Atago Co. Ltd., Tokyo, Japan) at 20 °C, with the results expressed as g kg^−1^ (mean ± SE) of fresh weight (FW). The TA was also determined in duplicate in the same juice by automatic titration (785 DMP Titrino, Metrohm, Burladingen, Germany) with 0.1 mol L^−1^ NaOH up to pH 8.10, using 1 mL diluted juice in 25 mL distilled H_2_O. The results (mean ± SE) were expressed as g malic acid equivalent kg^−1^ of FW.

### TPC and TAA of the hydrophilic and lipophilic fractions

On each sampling date, a subset of the 18 green peppers from each treatment was selected (six peppers from three replicates) and processed. The remaining portion of each pepper was then cut into small pieces, with the peduncle and seeds removed, and subsequently frozen in liquid nitrogen and stored at −80 °C until the functional and enzymatic analysis could be conducted. The TPC and TAA in both fractions (hydrophilic (H‐TAA) and lipophilic (L‐TAA)) were extracted and quantified in accordance with the methodology previously reported by Dobón‐Suárez *et al*.^26^ for ‘Lamuyo’ green pepper fruit. In summary, 10 mL of 50 mmol L^−1^ phosphate buffer (pH = 7.8) and 5 mL ethyl acetate were used to homogenise 5 g green pepper fruit. Subsequently, the extracts were subjected to centrifugation at 10 000 × *g* for 15 min at 4 °C. The TPC and H‐TAA were quantified using the lower fraction, while the L‐TAA was determined in the upper extract. The results (mean ± SE) were expressed as g kg^−1^ FW.

### Antioxidant–enzymatic system: ascorbate peroxidase, catalase and peroxidase activities

APX, CAT and peroxidase (POD) enzymes were analysed in freeze‐dried green pepper powder samples (flesh + skin tissues) stored at −80 °C. Samples were taken at harvest (day 0) and at regular intervals throughout the postharvest storage period (days 7, 14, 21 and 28). The activity of APX, CAT and POD were determined by homogenising 0.20 g of fine powder with 5 mL phosphate buffer (50 mmol L^−1^, pH 6.80) containing 1% (w/v) polyvinylpyrrolidone and 1 mmol L^−1^ ethylenediaminetetraacetic acid (EDTA). The resulting homogenate was subjected to centrifugation at 10 000 × *g* for 15 min at 4 °C. The supernatant was then employed in antioxidant enzyme assays in accordance with the methodology previously described by García‐Pastor *et al*.,^40^ with minor modifications. Antioxidant enzyme activities were expressed as units of enzymatic activity (U min^−1^ g^−1^) of dry weight (DW). One enzymatic unit (U) was defined as a 0.01 decrease of ascorbate at 290 and 240 nm min^−1^ for APX and CAT, respectively, and a 0.01 increase of absorbance at 470 nm min^−1^ for POD. The results are presented as the mean ± SE of three replicates (*n* = 3).

### Statistical analysis

All analytical determinations were subjected to statistical analysis, with three replicates (*n* = 3) being carried out for each parameter analysed. The results were expressed as the mean ± SE. The data were subjected to an analysis of variance (ANOVA). The sources of variation were the treatments and storage time. The mean comparisons were conducted using Tukey's honestly significant difference (HSD) test to ascertain whether the observed differences among the treatments or storage time were statistically significant. The resulting differences were represented with asterisks indicating *P < 0.05, **P < 0.01 and ***P < 0.001. No statistically significant differences were identified when the probability value (*P*) was equal to or greater than 0.05 and thus represented as NS. All analyses were conducted using the SPSS software package, version 17.0 for Windows (IBM Corporation, Armonk, NY, USA).

## RESULTS AND DISCUSSION

### Application of MeJA foliar spraying resulted in a notable increase in accumulated yield of green pepper plants during two growing seasons

**Figure 1:**
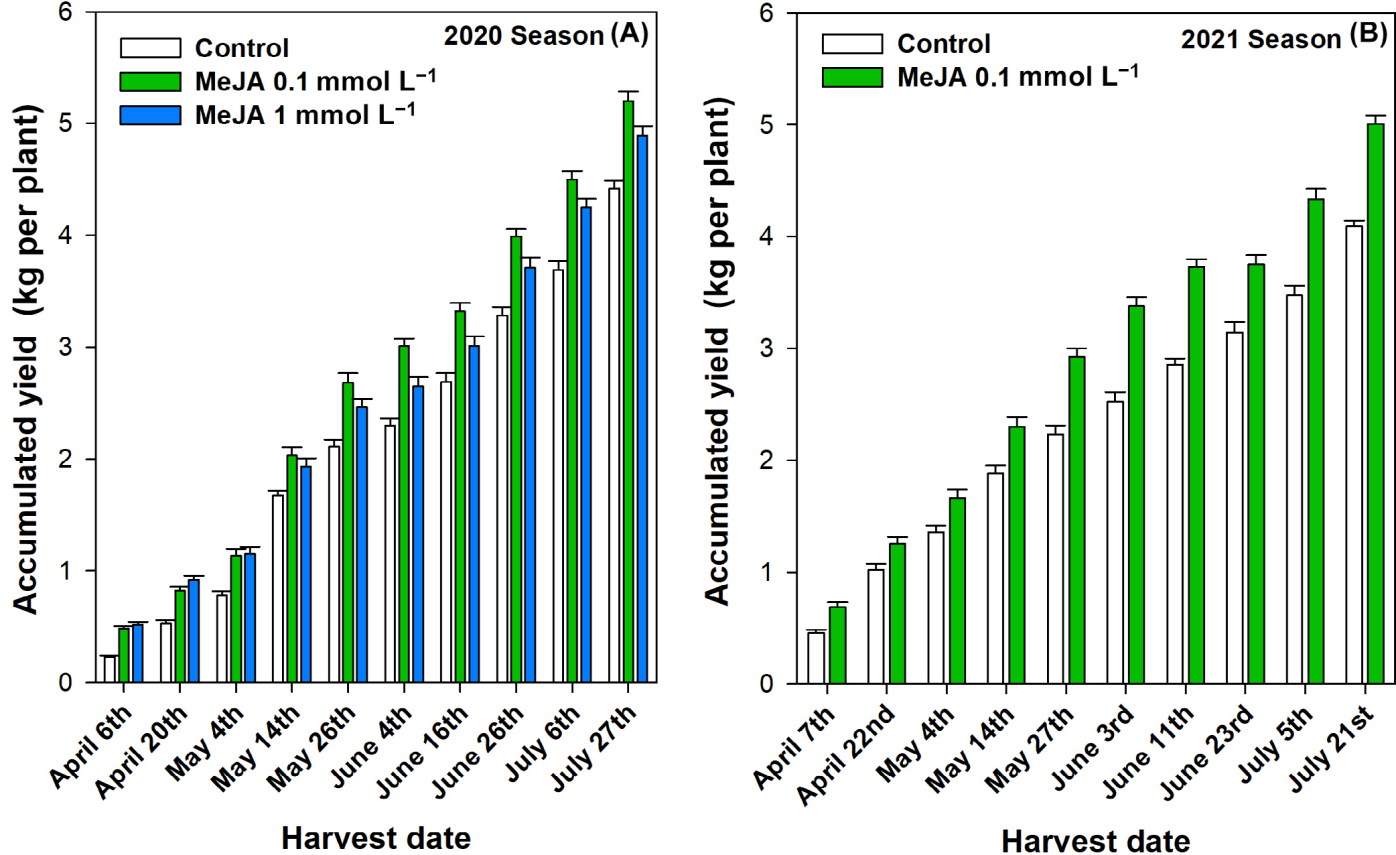
Accumulated yield (kg per plant) throughout the developmental and growth cycle of ‘Lamuyo’ green pepper plants, as affected by foliar spraying with methyl jasmonate (MeJA) at 0.1 and 1.0 mmol L^−1^ in the 2020 (A) and at 0.1 mmol L^−1^ in the 2021 (B) seasons. Data are the mean ± SE.

The accumulated yield of the green pepper crop was evaluated over two growing seasons (2020 and 2021) and expressed in kilograms per plant (Fig. 1(A),(B)). The results showed that the accumulated yield for both seasons was significantly higher in the MeJA‐treated plants than in the controls, with the first harvest showing a greater difference (Fig. 1 and Table 2). In the first season, preharvest MeJA treatments were applied, resulting in an improvement in yield per plant. The control plants had a total accumulated production of 4.42 ± 0.07 kg per plant. In contrast, the MeJA‐treated plants at 0.1 and 1.0 mmol L^−1^ reached a production of 5.20 ± 0.08 and 4.89 ± 0.09 kg, respectively. These values obtained in the MeJA‐treated plants represent a 0.78‐ and 0.47‐fold increase in total production compared to the untreated plants. However, the most effective treatment in terms of total yield increase was MeJA at 0.1 mmol L^−1^ (Fig. 1(A) and Table 2). This concentration was applied in the 2021 season to confirm the effect of the lowest dose of MeJA tested on the accumulative yield parameter. The plants previously treated with MeJA at 0.1 mmol L^−1^ showed a yield of 5.01 ± 0.07 kg per plant compared to 4.09 ± 0.054 kg per plant in the control plants (Fig. 1(B) and Table 2).

**Table 2:** ANOVA for crop yield, weight loss, respiration rate, physiochemical parameters and the antioxidant system using storage days and treatment as factors for each growing season (2020 and 2021)^†^

	Factor			
	2020 season		2021 season	
Parameter	Storage days	Treatment	Storage days	Treatment
Crop yield (kg per plant)		469.85*** MeJA 0.1 mmol L^−1^ = C MeJA 1.0 mmol L^−1^ = B Control = A		851.70*** MeJA 0.1 mmol L^−1^ Control
Weight loss (%)	275.81*** 7 = a 14 = b 21 = c 28 = d	12.15*** MeJA 0.1 mmol L^−1^ = A MeJA 1.0 mmol L^−1^ = A Control = B	1089.75*** 7 = a 14 = b 21 = c 28 = d	46.44*** MeJA 0.1 mmol L^−1^ Control
Respiration rate (mg CO_2_ kg^−1^ h^−1^)	1340.35*** 0 = c 7 = b 14 = bc 21 = bc 28 = a	10.33*** MeJA 0.1 mmol L^−1^ = A MeJA 1.0 mmol L^−1^ = A Control = B	953.66*** 0 = d 7 = c 14 = bc 21 = ab 28 = a	40.30*** MeJA 0.1 mmol L^−1^ Control
Firmness (N mm^−1^)	79.31*** 0 = d 7 = c 14 = b 21 = a 28 = a	5.82** MeJA 0.1 mmol L^−1^ = B MeJA 1.0 mmol L^−1^ = B Control = A	43.30*** 0 = c 7 = c 14 = b 21 = b 28 = a	31.20*** MeJA 0.1 mmol L^−1^ Control
Colour (hue°)	28.26*** 0 = c 7 = c 14 = b 21 = b 28 = a	54.41*** MeJA 0.1 mmol L^−1^ = A MeJA 1.0 mmol L^−1^ = B Control = C	30.15*** 0 = c 7 = b 14 = b 21 = b 28 = a	8.57** MeJA 0.1 mmol L^−1^ Control
TSS (g kg^−1^ FW)	89.13*** 0 = a 7 = b 14 = c 21 = d 28 = e	47.86*** MeJA 0.1 mmol L^−1^ = C MeJA 1.0 mmol L^−1^ = B Control = A	38.25*** 0 = a 7 = ab 14 = ab 21 = b 28 = c	30.25*** MeJA 0.1 mmol L^−1^ Control
TA (g kg^−1^ FW)	21.36*** 0 = c 7 = c 14 = b 21 = b 28 = a	2.36 NS MeJA 0.1 mmol L^−1^ = A MeJA 1.0 mmol L^−1^ = A Control = A	92.26*** 0 = d 7 = c 14 = b 21 = b 28 = a	15.64*** MeJA 0.1 mmol L^−1^ Control
TPC (g kg^−1^ FW)	153.39*** 0 = a 7 = b 14 = c 21 = d 28 = e	37.07*** MeJA 0.1 mmol L^−1^ = C MeJA 1.0 mmol L^−1^ = B Control = A	44.26*** 0 = a 7 = b 14 = c 21 = d 28 = d	54.81*** MeJA 0.1 mmol L^−1^ Control
H‐TAA (g kg^−1^ FW)	1318.77*** 0 = a 7 = b 14 = c 21 = d 28 = e	122.98*** MeJA 0.1 mmol L^−1^ = C MeJA 1.0 mmol L^−1^ = B Control = A	191.97*** 0 = a 7 = b 14 = b 21 = c 28 = d	139.07*** MeJA 0.1 mmol L^−1^ Control
L‐TAA (g kg^−1^ FW)	119.03*** 0 = a 7 = b 14 = c 21 = d 28 = e	79.89*** MeJA 0.1 mmol L^−1^ = B MeJA 1.0 mmol L^−1^ = A Control = A	284.32*** 0 = a 7 = b 14 = b 21 = c 28 = d	236.90*** MeJA 0.1 mmol L^−1^ Control
APX (U min^−1^ g^−1^ DW)	114.04*** 0 = b 7 = c 14 = c 21 = b 28 = a	295.56*** MeJA 0.1 mmol L^−1^ = C MeJA 1.0 mmol L^−1^ = B Control = A	39.89*** 0 = b 7 = c 14 = c 21 = b 28 = a	2020.08*** MeJA 0.1 mmol L^−1^ Control
CAT (U min^−1^ g^−1^ DW)	11.74*** 0 = b 7 = a 14 = b 21 = b 28 = c	210.56*** MeJA 0.1 mmol L^−1^ = B MeJA 1.0 mmol L^−1^ = C Control = A	17.04*** 0 = a 7 = b 14 = b 21 = b 28 = b	7241.87*** MeJA 0.1 mmol L^−1^ Control
POD (U min^−1^ g^−1^ DW)	66.40*** 0 = b 7 = cd 14 = d 21 = c 28 = a	60.87*** MeJA 0.1 mmol L^−1^ = C MeJA 1.0 mmol L^−1^ = B Control = A	63.01*** 0 = b 7 = c 14 = c 21 = b 28 = a	1466.79*** MeJA 0.1 mmol L^−1^ Control

TSS, total soluble solids; TA, total acidity; TPC, total phenolic content; H‐TAA, hydrophilic–total antioxidant activity; L‐TAA; lipophilic–total antioxidant activity; CAT, catalase; APX, ascorbate peroxidase; POD, peroxidase.

^†^ NS, not significant; asterisks indicate significance at *P < 0.05, **P < 0.01 and ***P < 0.001; data were previously tested for normality test. Different lower‐case letters indicate significant differences among storage days for each parameter and growing season tested. Upper‐case letters show significant differences among treatments for each parameter in the 2020 season.

In both growing seasons, the MeJA‐treated and control peppers were grown in a commercial greenhouse under similar climatic conditions and agronomic treatments, so the differences between the control and treated plants were only due to the preharvest treatments tested. A total of seven applications and ten harvests were carried out continuously from the beginning of April to the end of July, and the results for accumulated yield (kg per plant) for each treatment studied are shown (Table 1 and Fig. 1). The increase in accumulated yield was significantly higher (*P* < 0.001) in MeJA‐treated plants than in the control in both growing seasons, with 0.1 mmol L^−1^ concentration being the most effective in improving this parameter (Table 2). In addition, there is no information on the effect of MeJA fumigation on the total yield of pepper plants; therefore, for the first time, results are reported on the effect of MeJA on the yield of ‘Lamuyo’ pepper plants, depending on the concentration applied. To the best of our knowledge, the lowest concentration of MeJA tested in the present experiment (0.1 mmol L^−1^) could be a useful tool to increase the yield of green pepper plants grown under greenhouse conditions.

The application of MeJA has been demonstrated to enhance plant resistance to abiotic stress by triggering a cascade of physiological and metabolic changes that reduce oxidative damage by increasing antioxidant enzyme activity.^41^ A recent study demonstrated that pepper (*Capsicum annuum* L.) seedlings sprayed with 0.2 mmol L^−1^ MeJA exhibited a significantly increased dry matter mass compared to the untreated controls under low‐temperature/low‐light (LL) stress.^35^ This finding is consistent with the observation made by Yu *et al*.^42^ that plants with greater tolerance exhibited higher photosynthetic quantum yield. This was found to be correlated with the results of exogenous MeJA pretreatment of the photosystem II (PSII) reaction centre, which demonstrated an increase in the quantum yield of photosynthetic electron transfer, a decrease in the quantum yield due to heat dissipation and a decrease in the non‐regulated quantum yield. PSII, one of the most susceptible components of photosynthesis, results in a considerable decline in the electron transport chain under abiotic stress.^43^ This indicates that MeJA regulates PSII quantum yield and alleviates photoinhibition in pepper seedlings under LL conditions. Furthermore, the MeJA treatment resulted in an increase in photochemical efficiency, electron transport flux and PSII quantum efficiency to PSI, which serves as an important indicator for the assessment of plant health under conditions of adversity.^44^ Nevertheless, Kurowska *et al*.^45^ reported that an exogenous treatment with a high dose of MeJA (0.5 mmol L^−1^ for 120 h) resulted in a reduction in photosynthetic efficiency in barley seedlings. This was attributed to a decline in PSII parameters, which was associated with the downregulation of the *HvPsbR* gene, which encodes one of the extrinsic oxygen‐evolving complex proteins. Other studies have also reported that the application of higher doses of MeJA, such as 5, 10 and 20 mmol L^−1^, has a negative impact on crop yield, significantly decreasing fruit weight and size while also delaying the ripening process.^32^, ^46^

In the present experiment, MeJA fumigation was conducted prior to onset of the flowering stage. The elicitation process was maintained throughout the development and growth cycle of the fruit, spanning from February to July. This was achieved through the application of the elicitor approximately every 21 days in both growing seasons (Table 1). In addition to alleviating some negative effects on crop yield caused by biotic or abiotic stress, the elicitor MeJA may also increase the accumulated yield per plant by directly affecting the flowering rate, fruit set rate or abscission of pepper fruits from the plants, which occurs naturally during the fruit development process. Furthermore, an increase in Rubisco activity has been reported in previous studies on pepper fruits treated; other elicitors, including salicylic acid (SA),^26^, ^47^, ^48^, ^49^, ^50^ have also demonstrated similar results. Similar results regarding an increase in yield have been reported in other fruit species with lower concentrations of MeJA as a preharvest treatment (1, 0.5, 0.25 and 0.1 mmol L^−1^) in plum, artichoke, table grape, pomegranate fruit and tomato. These findings are supported by the studies of Martínez‐Esplá *et al*.,^36^, ^51^ García‐Pastor *et al*.^32^, ^37^ and Baek *et al*.,^52^ respectively, which indicate that MeJA may be a dose‐dependent compound.

### Application of MeJA foliar spraying effectively delays the deterioration in physiochemical quality of green pepper fruit after prolonged storage at optimal temperatures

**Figure 2:**
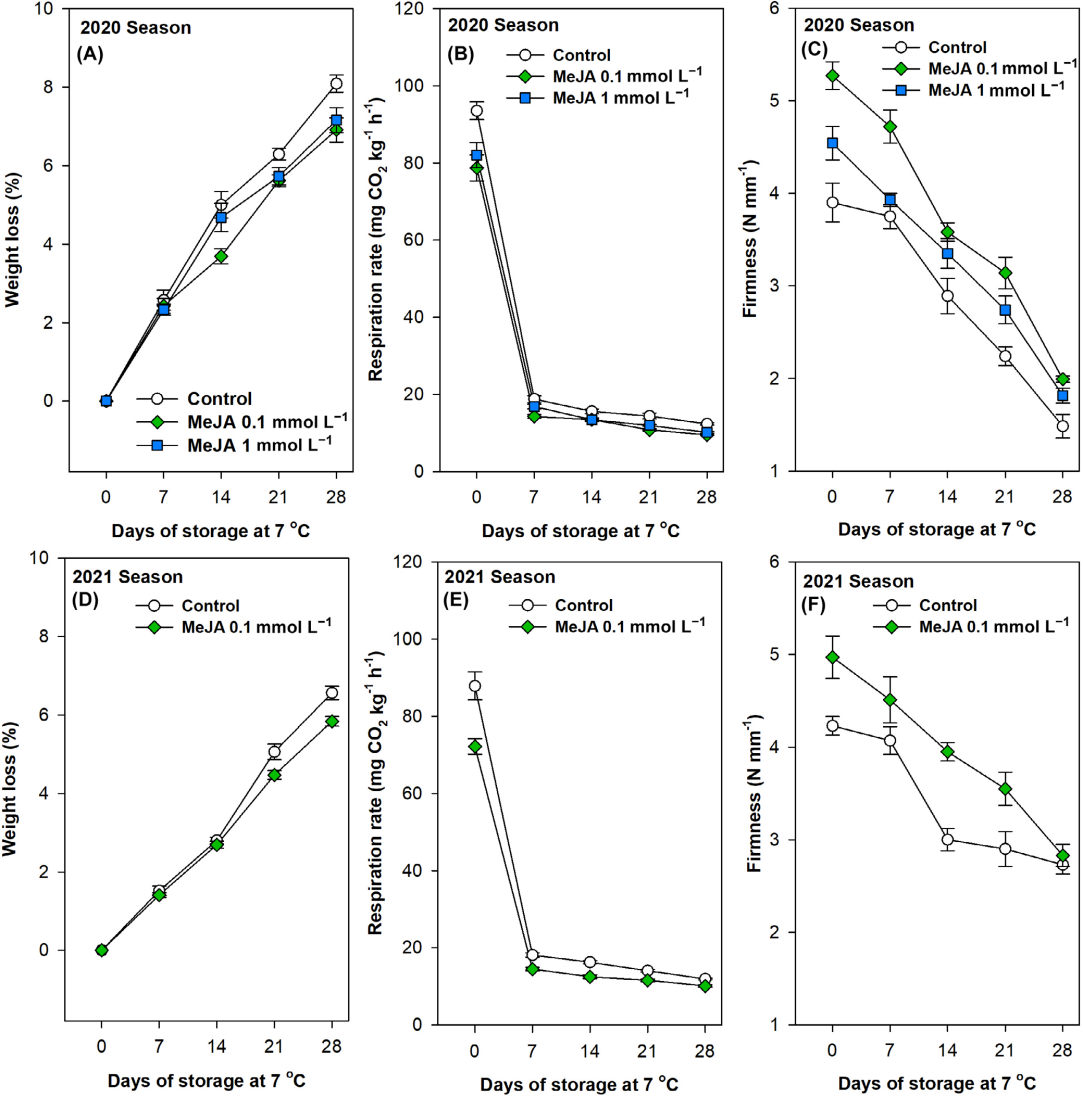
Effects of preharvest methyl jasmonate (MeJA) treatments at 0.1 and 1.0 mmol L^−1^ in the 2020 (A,B,C) and at 0.1 mmol L^−1^ in the 2021 (D,E,F) seasons on weight loss (%; A,D), respiration rate (mg CO_2_ kg^−1^ h^−1^; B,E) and firmness (N mm^−1^; C,F) of ‘Lamuyo’ green pepper fruit during 28 days of cold storage at 7 °C. Data are the mean ± SE.

In the present study, ‘Lamuyo’ bell pepper plants were subjected to seven fumigation applications with MeJA solutions at concentrations of 0.1 and 1.0 mmol L^−1^ during the 2020 season, and 0.1 mmol L^−1^ during the 2021 season (Table 1). A number of fruit quality parameters were evaluated at harvest and subsequently during the storage period. There was a significant increase (*P* < 0.001) in fruit weight loss over time in both growing seasons (Fig. 2(A),(D) and Table 2). The rate of this increase exhibited significant variation contingent on the foliar spraying treatment in both seasons (*P* < 0.001; Table 2).

Fruits of the control treatment exhibited the highest rate of weight loss, while the lowest rate was observed in the MeJA‐treated pepper fruits, which demonstrated a 1.17‐ and 1.12‐fold reduction at the end of the storage period in the 2020 and 2021 seasons, respectively (Fig. 2(A),(D)). However, no significant differences were observed between the two concentrations of MeJA tested (Table 2). Therefore, the foliar spraying application of MeJA at 0.1 and 1.0 mmol L^−1^ effectively delayed the weight loss after 28 days of storage at 7 °C compared with the control (Fig. 2(A)), with these results being confirmed for the 2021 growing season (Fig. 2(D)). Conversely, the respiration rate demonstrated a significantly (*P* < 0.001) reduced pattern from harvest until 28 storage days at optimal temperature in both growing seasons (Fig. 2(B),(E) and Table 2). The application of MeJA via foliar spraying at concentrations of 0.1 and 1.0 mmol L^−1^ resulted in a significant (*P* < 0.001) reduction in the respiration rate in comparison to the control, although no significant differences were observed between the two concentrations during the 2020 season (Fig. 2(B) and Table 2). The same reduction in behaviour was observed for 0.1 mmol L^−1^ MeJA treatment in the 2021 season, demonstrating a significant (*P* < 0.001) impact of this dose on the minimization of this indicator of fruit metabolic activity at both the harvest and 28‐day storage period (Fig. 2(E) and Table 2). A significant decline (*P* < 0.001) in fruit firmness was observed during the storage period, with a particularly pronounced rate of decline evident in the 2020 growing season (Fig. 2(C),(F) and Table 2). In this season, green pepper fruit fumigated with MeJA at 0.1 and 1.0 mmol L^−1^ exhibited significantly higher firmness (*P* < 0.01; 26% and 14%, respectively) than the control at harvest (Fig. 2(C) and Table 2). This effect was maintained during the storage period, although no significant differences were observed between the two doses (Table 2). Furthermore, a delay of softening was observed in green pepper fruits treated with MeJA at 0.1 mmol L^−1^ during the 2021 season. This treatment significantly increased firmness levels (*P* < 0.001) compared to control, except at 28 days (Fig. 2(F) and Table 2).

**Figure 3:**
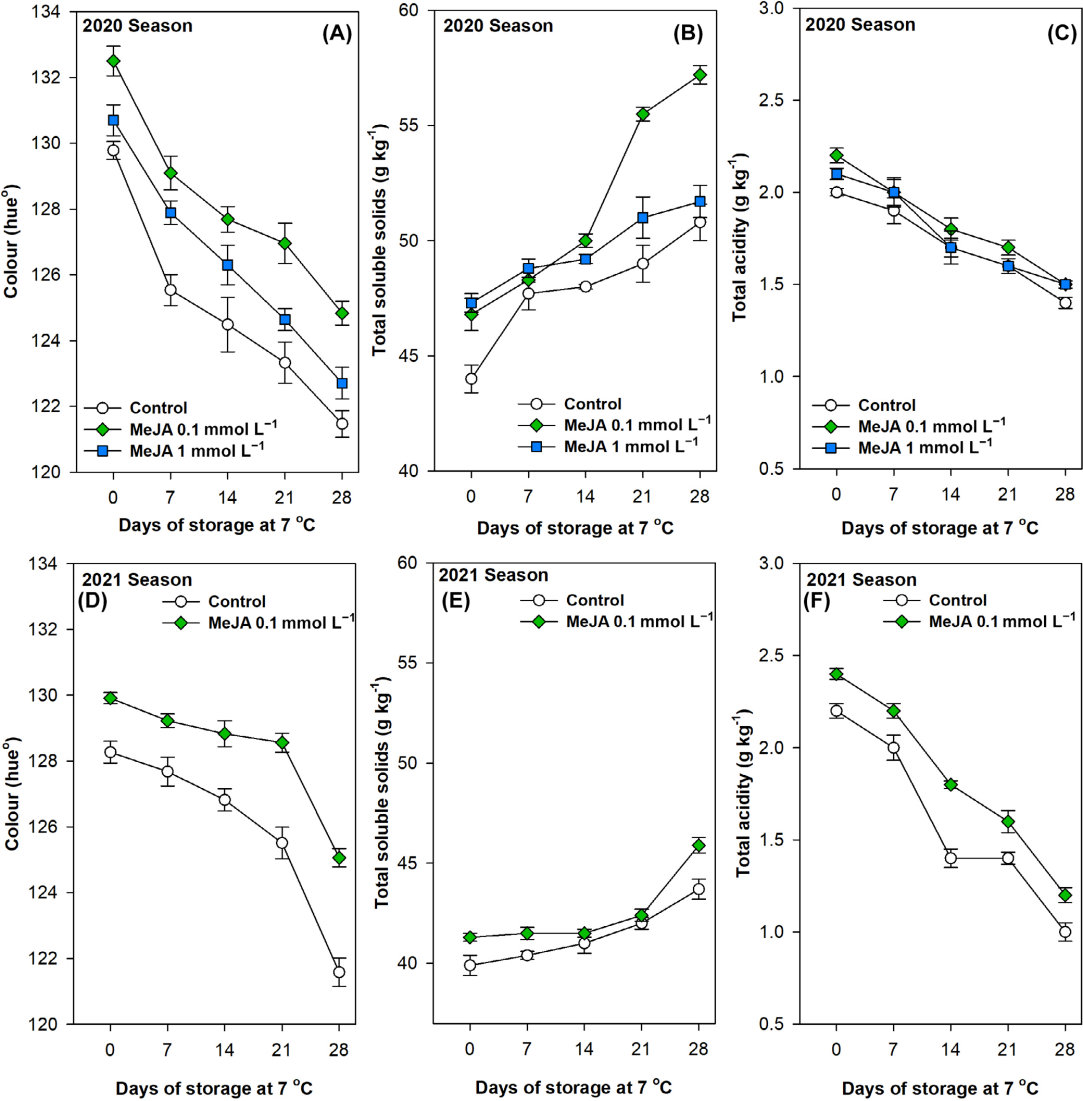
Effects of preharvest methyl jasmonate (MeJA) treatments at 0.1 and 1.0 mmol L^−1^ in the 2020 (A,B,C) and at 0.1 mmol L^−1^ in the 2021 (D,E,F) seasons on colour (hue°; A,D), total soluble solids (g kg^−1^; B,E) and total acidity (g kg^−1^; C,F) of ‘Lamuyo’ green pepper fruit during 28 days of cold storage at 7 °C. Data are the mean ± SE.

The green colour of pepper fruits, expressed as hue angle, demonstrated a significant (*P* < 0.001) decline over the 28‐day postharvest storage period at 7 °C, across both seasons, for all treatments tested (Fig. 3(A),(D) and Table 2). In the 2020 season, this physical parameter was significantly (*P* < 0.001) higher in those pepper fruits treated with MeJA compared with untreated peppers, particularly for the 0.1 mmol L^−1^ concentration (Fig. 3(A) and Table 2). In the subsequent season, comparable outcomes were documented, with the 0.1 mmol L^−1^ MeJA fumigation demonstrating a statistically significant (*P* < 0.01) elevation in hue angle values in comparison to the control fruits at harvest and throughout the postharvest storage period (Fig. 3(D) and Table 2). The content of TSS demonstrated a significant (*P* < 0.001) increase during the cold storage period for both growing seasons under study (Fig. 3(B),(E) and Table 2). The green pepper fruits harvested from plants treated with MeJA exhibited a significantly (*P* < 0.001) higher content of TSS at harvest and after 28 days of cold storage, particularly at 0.1 mmol L^−1^ concentration (Fig. 3(B),(E) and Table 2). However, the accumulation of these values was more pronounced in the 2020 season. Conversely, TA exhibited a notable (*P* < 0.001) decline across all treatments throughout the postharvest storage period in both seasons (Fig. 3(C),(F) and Table 2). In the 2020 season, there were no significant differences (*P* ≥ 0.05) in TA content between MeJA‐treated and untreated green pepper fruits throughout the 28‐day storage period (Fig. 3(C) and Table 2). However, in the 2021 season, TA was significantly (*P* < 0.001) higher in fruits treated with 0.1 mmol L^−1^ MeJA, and this difference was maintained throughout the experiment (Fig. 3(F) and Table 2).

Bell peppers are a highly perishable vegetable, exhibiting a limited shelf‐life and a high susceptibility to disease. It is crucial to emphasise the importance of appropriate handling and adequate care to maintain postharvest quality.^14^, ^53^ The primary factors that negatively impact the postharvest quality of peppers during transportation, short‐term storage, and marketing and sales are water loss, softening and chilling injury.^15^, ^16^, ^54^ Other factors that contribute to postharvest deterioration include physiological disorders, disease and mechanical damage.^55^ The fruit is susceptible to several adverse effects, including flaccidity, wilting, shrivelling, fungal infections and deterioration, which collectively contribute to its relatively short shelf‐life.^17^ Such factors frequently impact consumer acceptance of the fruit. It is therefore imperative to enhance the shelf‐life of bell peppers to reduce postharvest losses and to enhance food security and sustainability.

A number of metabolic and physical characteristics associated with fruit quality were evaluated at harvest and throughout the subsequent storage period. The findings revealed that preharvest fumigation with MeJA significantly delayed weight loss, respiration rate and softening of pepper fruit during a 28‐day storage period at 7 °C (Fig. 2). To the best of our knowledge, this is the inaugural report on the impact of diverse preharvest MeJA applications on the quality parameters of bell peppers (*Capsicum annuum* L.) during postharvest storage. The findings provide insights into the underlying mechanisms of hormonal field application and its influence on the storability of pepper fruits. The preharvest application of MeJA has a significant impact on fruit ripening, with the effect varying according to the concentration used, in both climacteric and non‐climacteric fruits. The loss of water in sweet peppers is a significant issue that arises during storage (Fig. 2(A),(D)), mainly caused by transpiration through fruit skin and leading to alterations in fruit firmness and a decline in quality.^56^ Previous reports indicate that MeJA applications during the postharvest period of different fruits have an impact on the fruit weight loss in treated blueberries,^57^ apricots,^58^ oranges^59^ and strawberries.^60^ Furthermore, recent studies have indicated that the fumigation of MeJA can significantly delay losses in fruit weight during prolonged storage periods, thereby maintaining the quality of pomegranates, strawberries, lemons and blackberries.^36^, ^37^, ^61^, ^62^, ^63^ This is associated with the maintenance of cell integrity.^62^ MeJA, an ester of jasmonic acid, has been demonstrated to activate an antioxidant defence mechanism against free radicals and retard membrane peroxidation.^64^ This could be the reason for the reduced weight loss previously reported in raspberries.^63^ Conversely, Karaman *et al*.^65^ reported that the preharvest application of MeJA significantly increased weight loss of plum at the end of the storage period. This phenomenon can be attributed to the fact that MeJA causes the emission of ethylene, a gaseous hormone involved in plant development and ripening, in some species of vegetables, particularly at specific developmental stages.

The results of the experiments indicate that the treatments resulted in a notable decline in fruit respiration rate (Fig. 2(B),(E)) and fruit softening (Fig. 2(C),(F)). This reduction in respiration and the observed loss of weight (Fig. 2(A),(D)) in treated green peppers are also likely attributed to these effects. The relationship between respiratory rate and MeJA treatment appears to be dependent on the species in question. For instance, González‐Aguilar *et al*.^66^ observed that MeJA had no effect on the respiration rate of mango, whereas Pérez *et al*.^67^ noted the opposite to be true in strawberries. Firmness is associated with cell turgidity and the thickness of the skin, and it is a crucial factor in determining commercial acceptance. The major cell wall components are pectins, hemicelluloses and cellulose. The process of fruit softening is directly related to the depolymerization of these components by cell wall hydrolytic enzymes, which include polygalacturonases, pectin methyl esterases and cellulases. Therefore, the higher firmness levels at harvest and the lower softening of MeJA‐treated green pepper fruit may be attributed to reduced activity of these cell wall‐degrading enzymes, particularly pectinases, as previously observed in MeJA postharvest‐treated longkong and mango fruit.^68^, ^69^ Other authors have similarly observed a reduction in weight loss and maintenance of firmness in lemon, peach and tomato fruit as a result of preharvest MeJA treatments. This phenomenon has been linked with the preservation of cellular integrity and a reduction in fruit respiration rates.^52^, ^62^, ^70^, ^71^ Furthermore, elevated phenylalanine ammonia‐lyase (PAL) activity regulates lignin deposition in the cell wall, contributing to enhanced fruit firmness during ripening and low‐temperature storage.^72^ Previous studies have demonstrated that the application of exogenous MeJA upregulated phenolic metabolism by increasing PAL activity during postharvest storage of strawberries,^73^ tomatoes,^74^ sweet cherries^75^ and raspberries.^63^ In addition, the application of MeJA in peach fruit has been demonstrated to enhance the activity of the POD enzyme, which plays a role in lignin biosynthesis.^76^ This, in turn, may contribute to an increase in fruit firmness. Indeed, Zhang *et al*.^77^ have recently reported that preharvest MeJA treatment had an activating effect on the secondary metabolic pathway, upregulating the most differentially expressed genes in this pathway in postharvest berries. This will be discussed in further detail below. Therefore, the preservation of fruit firmness through the restriction of weight loss and respiration rate represents an additional mechanism by which the fumigation of MeJA treatment prolongs the postharvest storability of bell pepper fruits. Further studies focused on the effect on the activity of cell wall hydrolytic enzymes could be conducted.

The quality of pepper fruit is contingent upon a multitude of intrinsic characteristics, encompassing aspects of visual presentation, flavour, chemical composition and nutritional value. Among these quality traits, the colour of pepper fruit is one of the most intuitive and essential quality traits, influencing consumer and breeder preferences during the purchase and cultivation process. The light‐ and dark‐green colouration of immature peppers is associated with the chlorophyll content of the fruits. The green colour of pepper fruit, as indicated by the hue angle, was maintained during postharvest storage at 7 °C in those fruits that had been fumigated with MeJA (Fig. 3(A),(D)). This could be related to a delay in chlorophyll degradation. Similarly, Bron *et al*.^78^ also reported a slight retention of skin colour in papaya fruits dipped in 0.01 mmol L^−1^ MeJA solutions. Other research has investigated the impact of varying concentrations of MeJA postharvest treatment on chlorophyll degradation in apple fruit. The findings indicate that exogenous 0.01 mmol L^−1^ MeJA delayed degreening and ripening in apple fruit, whereas a 1.5 mmol L^−1^ MeJA treatment had the opposite effect.^79^ In a similar vein, other reports have indicated that MeJA treatments have been observed to increase the red peel colour development of apples in comparison to the control treatment.^46^, ^80^ Prior research has demonstrated that plum, mango and raspberry fruit treated with MeJA display elevated hue angle values.^36^, ^63^, ^81^, ^82^ This may be attributed to delayed colour development and anthocyanin degradation, as well as higher levels of flavonoids. This phenomenon is associated with the oxidation of flavonoids and anthocyanins by free radicals during storage, which results in premature ageing and alterations in fruit colour attributes.

The green pepper fruits harvested from plants fumigated with MeJA exhibited a higher content of TSS (Fig. 3(B),(E)) and TA (Fig. 3(C),(F)) at harvest in both growing seasons. Furthermore, these higher contents were maintained throughout the postharvest period, in comparison to the untreated fruits. Therefore, preharvest MeJA fumigation resulted in both an enhancement of green pepper organoleptic quality parameters, including firmness, green colour, TSS and TA, and a delay of quality losses during postharvest. Consequently, the application of MeJA at 0.01 or 0.1 mmol L^−1^ in blackberry cultivars has been observed to increase the content of TSS, with the effect being proportional to the applied concentration.^83^ In mango, preharvest MeJA treatment resulted in fruit with increased concentrations of glucose, fructose and sucrose.^82^ The impact of MeJA treatments on elevating fruit TSS and sugar content can be attributed to an enhancement in the net photosynthetic rate of ‘Lamuyo’ pepper plants, in addition to an increase in starch degradation and the production of fructose and glucose. In this regard, it has been demonstrated that MeJA at 1.0 mmol L^−1^ stimulates dry matter accumulation in cauliflower seedlings by promoting chlorophyll synthesis and increasing the net photosynthetic rate, stomatal conductance and intercellular CO_2_ concentration.^84^ Therefore, the application of MeJA would result in an increase in available photoassimilates, thereby supporting the growth of pepper fruit. The increase in TSS of green pepper fruits treated with MeJA is consistent with previous findings in studies involving plums,^81^ lemon^62^ and yellow pitahaya.^85^ Furthermore, sugar accumulation is a crucial indicator of quality and is closely linked to the expression of genes involved in defence responses.^86^ Therefore, the elevated TSS observed in the treated groups may also be attributed to the crop's response to the stress induced by MeJA.^71^ Nevertheless, the mechanism of jasmonate‐associated sugar accumulation in pepper fruit remains to be elucidated.

With respect to TA, the observed decrease can be attributed to the utilization of organic acids during the respiration process or their conversion into sugars. The preharvest application of MeJA resulted in lemon fruit exhibiting elevated concentrations of individual sugars and organic acids in the flavedo and juice, both at harvest and following a 35‐day storage period.^62^ Consequently, the utilization of reduced doses of MeJA (0.1 and 0.01 mmol L^−1^) has been observed to result in elevated TSS and TA content in table grapes.^37^ In a previous study, the application of MeJA at concentrations of 0.1, 0.3, 0.5 and 0.7 mmol L^−1^ to Kinnow mandarin prior to harvest resulted in elevated fruit TA values and a reduced TSS/TA ratio (indicative of the ripening index). Of these concentrations, 0.5 mmol L^−1^ was identified as the most effective in delaying the ripening process.^87^ Similarly, Martínez‐Esplá *et al*.^36^ observed that acidity losses were delayed by 0.5 mmol L^−1^ MeJA preharvest treatments, while 1.0 and 2.0 mmol L^−1^ doses had no significant effect. Their findings also indicated that MeJA can differentially impact each of the parameters involved in fruit ripening, a hypothesis that has been previously proposed by Rudell *et al*.^46^ in apple fruit. From an agronomic and commercial standpoint, the results would be of significant importance, as green pepper fruit with enhanced firmness, TSS and TA would be more highly valued by consumers. The findings of past and present studies collectively indicate that a preharvest treatment with MeJA represents a promising strategy for enhancing the retention of quality traits in a range of horticultural crops after harvest. Further research is required to establish detailed relationships between jasmonates and the inhibition of senescence, and the effect of the plant growth regulator should be carefully considered on a species‐ and cultivar‐specific basis.

Future research on bell peppers could significantly enhance the mechanistic understanding of MeJA effects on quality. Regarding jasmonate‐associated sugar accumulation, the present study noted that this precise mechanism warrants further elucidation in bell pepper fruit. Insights from studies on other fruits, such as tomato, can guide this.^88^ For instance, exogenous MeJA in tomato was shown to significantly accelerate ripening, resulting in higher sucrose content and lower glucose and fructose levels. This was linked to enhanced sucrose phosphate synthase (SPS) activity and upregulation of sucrose biosynthesis genes (*SPS1*–*4* and *SPP2*), while simultaneously inhibiting acid invertase (AI) and neutral invertase (NI) activities and downregulating related degradation genes (*TIV1*, *Lin6*, *Lin7*, *Lin9*, *CIN6*).^88^ Therefore, future work in bell peppers should investigate these specific enzymatic activities and gene expressions to clarify the sugar accumulation mechanism. Concerning the preservation of firmness and the impact on cell wall hydrolytic enzymes, the present research suggests further studies on enzymes like polygalacturonases (PG) and pectin methyl esterases (PME). Research in raspberries has demonstrated that preharvest MeJA spray effectively reduced firmness loss by maintaining higher total pectin and protopectin levels, and notably lowering the activities of PME, PG and cellulase (Cx) enzymes.^63^ A direct investigation into the activity and gene expression of these cell wall‐degrading enzymes in MeJA‐treated bell peppers would provide crucial mechanistic links to the observed firmness retention.

### Application of MeJA foliar spraying positively modulated the secondary metabolism and reduced the oxidative stress in green pepper fruit

**Figure 4:**
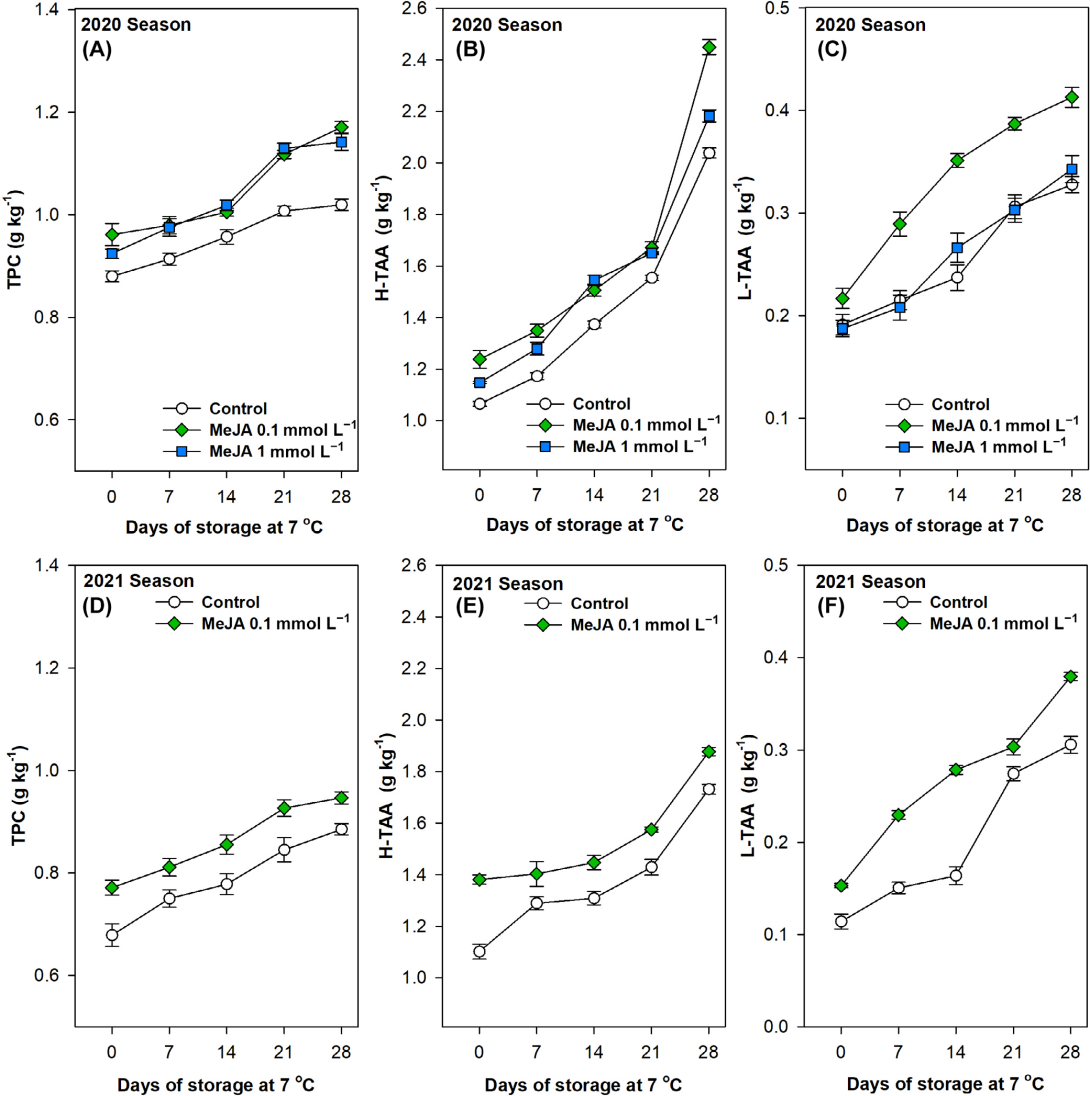
Effects of preharvest methyl jasmonate (MeJA) treatments at 0.1 and 1.0 mmol L^−1^ in the 2020 (A,B,C) and at 0.1 mmol L^−1^ in the 2021 (D, E,F) seasons on total phenolic content (TPC, g kg^−1^; A,D) and hydrophilic–total antioxidant activity (H‐TAA, g kg^−1^; B,E) and lipophilic–total antioxidant activity (L‐TAA, g kg^−1^; C,F) of ‘Lamuyo’ green pepper fruit during 28 days of cold storage at 7 °C. Data are the mean ± SE.

The parameters studied from the non‐enzymatic antioxidants system for controlling the free radicals, TPC and TAA, both in the H‐TAA and L‐TAA fractions, demonstrated a markedly increasing trend from harvest until 28 days of storage at 7 °C for all treatments tested (Fig. 4). This increase was statistically significant (*P* < 0.001; Table 2). The preharvest application of MeJA resulted in a statistically significant (*P* < 0.001) enhancement in TPC compared to the control fruits at the harvest, with the phenolic content maintained at a higher level throughout the postharvest period (Fig. 4(A) and Table 2), particularly for the 0.1 mmol L^−1^ concentration. In the 2021 season, comparable outcomes were documented, with MeJA demonstrating a statistically significant (*P* < 0.001) promotion of phenolic accumulation in green pepper fruits harvested from plants treated with 0.1 mmol L^−1^ MeJA (Fig. 4(D) and Table 2). Furthermore, a notable (*P* < 0.001) stimulation of both H‐TAA and L‐TAA was evident in green pepper fruits harvested from MeJA‐treated plants at harvest (Fig. 4(B),(C) and Table 2). Following prolonged storage at optimal temperatures, the TAA of both the hydrophilic and lipophilic fractions in the MeJA‐treated pepper fruits was maintained at higher levels than in the untreated fruits, with significant (*P* < 0.001) differences being reported (Fig. 4(B),(C) and Table 2). These favourable outcomes pertaining to the stimulation of H‐TAA and L‐TAA by MeJA foliar spraying were replicated in the 2021 growing season, with the same statistically significant differences being observed (Fig. 4(E),(F) and Table 2).

Total antioxidants play an essential role in increasing storability and maintaining fruit quality during the postharvest period.^89^ TPC contributes to the total non‐enzymatic antioxidant levels in fresh horticultural produce.^90^ It can be hypothesised that elevated levels of TPC in MeJA‐treated green pepper fruits may contribute to augmented total antioxidant levels in treated fruit compared to the control (Fig. 4). Phenolics represent one of the most significant secondary metabolites, playing a pivotal role in enhancing fruit quality and contributing to antioxidant reactions that initiate stress‐mediated defence mechanisms in plants.^90^ This study demonstrates, for the first time, that the applied dose of 0.1 mmol L^−1^ of MeJA is also an important factor affecting the quality development and secondary metabolism of ‘Lamuyo’ green bell pepper. Higher phenolic concentrations contribute to the maintenance of membrane integrity by mitigating the propagation of ROS, which in turn reduces lipid peroxidation. This results in a delayed onset of oxidative stress and improved fruit quality.^90^ It has been demonstrated that the application of MeJA prior to harvesting can result in elevated levels of phenolic compounds in a range of fruit crops. This phenomenon has been observed in plums,^36^, ^91^ apples,^81^ mangoes,^82^ table grapes,^32^ lemons,^39^ pomegranates,^37^, ^40^ sweet cherries,^38^, ^75^ strawberries,^73^ tomatoes^74^ and raspberries.^63^ The stimulation of the enzymatic activities and gene expressions involved in the phenylpropanoid pathway has been considered as a potential mechanism underlying these observations.^74^

During the postharvest storage period of green lilies, MeJA treatment was observed to significantly activate the expression of key genes involved in phenylpropane metabolism, including PAL, C4H and 4CL, and to increase their enzyme activity.^92^ Additionally, the TPC was found to be correlated with an enhancement of TAA.^62^ In this regard, Baek *et al*.^71^ observed that MeJA and SA treatments enhanced antioxidant activities at two harvesting stages and throughout the storage period, without affecting the sensory qualities of tomato. Conversely, postharvest MeJA treatments resulted in a reduction in total phenolics and antioxidant activity in carambola.^93^ Additionally, the application of MeJA has been documented to enhance the synthesis of specific flavonoids. However, Wang *et al*.^94^ observed that the changes in phenolic content were typically greater than those observed in flavonoid content. For example, the content of phenolics was increased by 25%, while the content of flavonoids was only increased by 8% in pomegranates treated with 0.1 mmol L^−1^.^95^ It can therefore be concluded that the fumigation of bell pepper plants with MeJA could be an effective strategy for significantly activating the non‐enzymatic antioxidant system. However, the effect on the underlying regulatory mechanism has yet to be reported. It would be beneficial for future studies to address the regulation of the relative expression of the majority of genes involved in the phenylpropane metabolism following the application of MeJA foliar spraying. On the other hand, MeJA treatment significantly enhances the H‐TAA in bell peppers, primarily driven by increased vitamin C content. This potent antioxidant, also known as ascorbic acid, contributes substantially to the fruit's overall antioxidant capacity, as evidenced by its strong correlation with total polyphenol and reducing power.^96^ Beyond vitamin C, MeJA stimulates antioxidant enzymes and elevates other beneficial compounds like phenolics, flavonoids, capsaicinoids and carotenoids.^96^, ^97^, ^98^ Consequently, preharvest MeJA application collectively improves the nutritional value and health benefits of bell peppers.^99^

**Figure 5:**
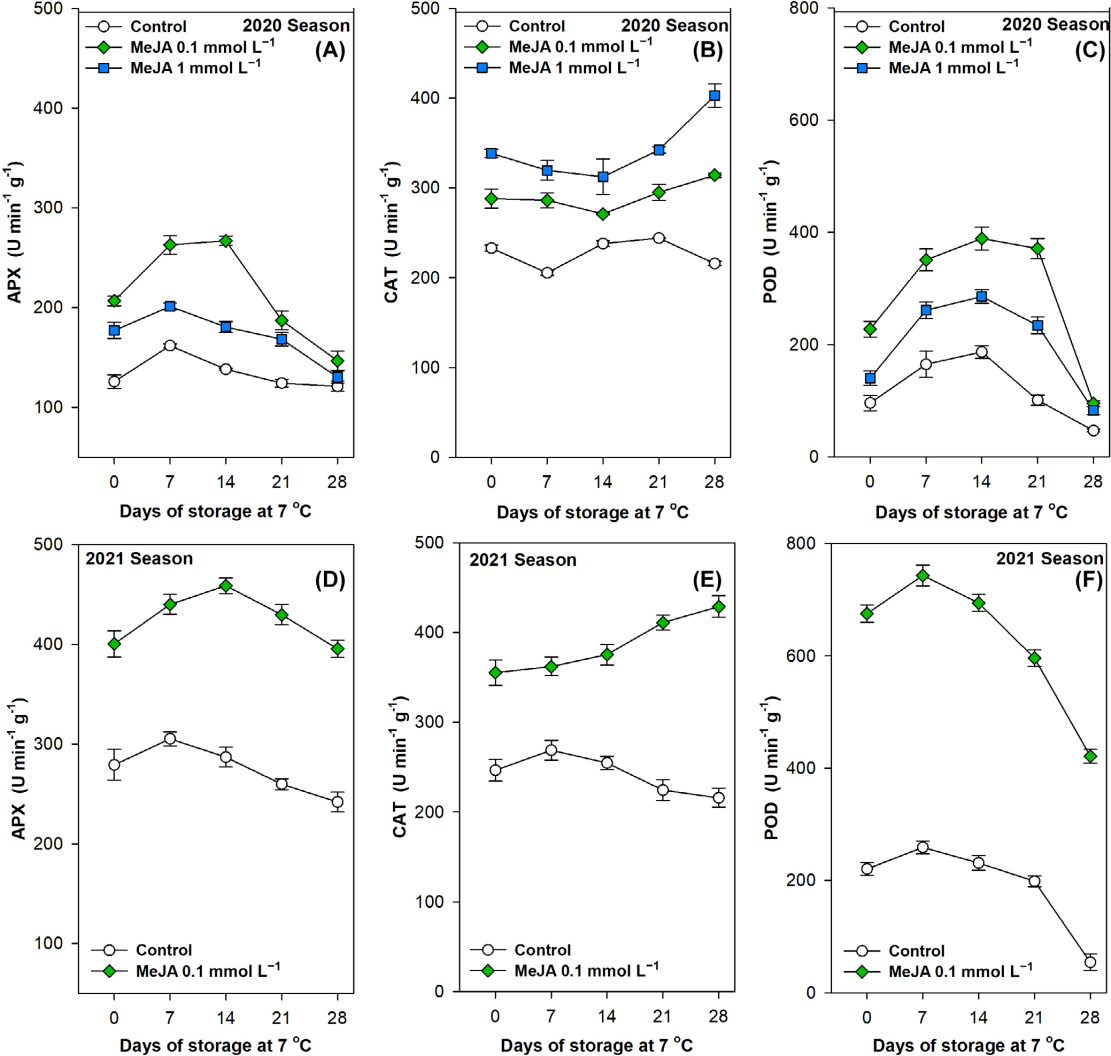
Effects of preharvest methyl jasmonate (MeJA) treatments at 0.1 and 1.0 mmol L^−1^ in the 2020 (A, B,C) and at 0.1 mmol L^−1^ in the 2021 (D, E,F) seasons on the activity of ascorbate peroxidase (APX, U min^−1^ g^−1^; A,D), catalase (CAT, U min^−1^ g^−1^; B,E) and peroxidase (POD, U min^−1^ g^−1^; C,F) of ‘Lamuyo’ green pepper fruit during 28 days of cold storage at 7 °C. Data are the mean ± SE.

With regard to the antioxidant enzymatic system, the activities of APX, CAT, and POD were examined (Fig. 5). The three enzymes exhibited a statistically significant (*P* < 0.001) variation in their values throughout the postharvest storage period at 7 °C for all treatments tested, with a notable decline observed after 14 days (Fig. 5 and Table 2). The application of MeJA resulted in a notable improvement in the activities of APX (Fig. 5(A),(D)), CAT (Fig. 5(B),(E)) and POD (Fig. 5(C),(F)) at harvest. Furthermore, the activities of these enzymes remained significantly elevated (*P* < 0.001) in comparison to the control in both the 2020 and 2021 seasons (Table 2). The 0.1 mmol L^−1^ dose proved to be the most efficacious in stimulating the activity of these antioxidant enzymes (Fig. 5 and Table 2). It is well established that the production of ROS in plant cells is increased during the postharvest ripening process as a consequence of normal metabolic processes. These ROS are then eliminated by enzymatic systems. Antioxidant enzymes are involved in the radical scavenging of ROS, thereby acting as a mechanism for repairing cell oxidative damage.^1^ In eggplant, MeJA has been demonstrated to promote antioxidant enzyme activity and an increase in the relative expression of their corresponding genes, thereby enhancing the plant's protective effect against oxidative stress.^100^ Similar outcomes were corroborated in other fruit species, including plums,^91^ table grapes,^32^ pomegranates^37^ and lemons.^62^ These findings have led to an extension of the fruit shelf‐life. The increase of these enzymes may enhance the tissue capacity to eliminate ROS, thereby delaying the ripening and senescence processes discussed in the previous section. Ultimately, the foliar application of MeJA to bell pepper plants demonstrated a favourable modulation of the secondary metabolism of green pepper fruits, which could be associated with the postponement of physiochemical quality deterioration during the postharvest ripening and senescence process at 7 °C.

To enhance the understanding of how MeJA modulates secondary metabolism and the antioxidant system in bell peppers, future work should delve into molecular regulatory mechanisms. The present study reported that MeJA treatments increased phenolic content and total antioxidant activity by stimulating enzymes like APX, CAT and POD, suggesting a link to the phenylpropanoid pathway. Studies in kiwifruit offer a detailed roadmap for this investigation, showing that MeJA enhanced the activity of key phenylpropanoid pathway enzymes such as PAL, C4H and 4CL, while also upregulating the expression of their corresponding genes (*AcPAL*, *AcC4H*, *Ac4CL*).^101^ This led to increased accumulation of total phenolics and flavonoids.^101^ Similarly, in raspberries, MeJA application was found to upregulate the phenylpropanoid pathway, resulting in higher endogenous phenolics and activities of PAL and shikimate dehydrogenase.^63^, ^102^ Both kiwifruit and raspberry studies also consistently showed that MeJA enhanced antioxidant enzyme activities (e.g., POD, CAT, SOD) and reduced oxidative damage.^63^, ^101^ Additionally, higher PAL activity regulates lignin deposition in cell wall, contributing to higher fruit firmness during ripening and low‐temperature storage.^72^ Therefore, future research on bell peppers should prioritise assessing the relative expression of these key genes (PAL, C4H, 4CL) and analysing the activities of other relevant antioxidant enzymes to fully elucidate the molecular pathways activated by preharvest MeJA, thereby enhancing health benefits upon consumption.

The effect of MeJA dosage on fruit quality is species‐dependent.^103^, ^104^, ^105^, ^106^ In litchi, a 2 mmol L^−1^ MeJA vacuum infiltration significantly reduced pericarp browning and weight loss, retaining higher total soluble solids, titratable acidity, ascorbic acid, phenolics and anthocyanins, while inhibiting reactive oxygen species production.^103^ For apricots, 1.0 mmol L^−1^ MeJA (MeJA2) with modified atmosphere packaging (MAP) improved TPC and antioxidant capacity, and significantly increased vitamin C, while 0.5 mmol L^−1^ MeJA (MeJA1) with MAP reduced weight loss and respiration rates, delaying softening.^104^ Preharvest application of 2 mmol L^−1^ MeJA to sweet cherry significantly reduced fruit cracking and improved red color, phenolics, flavonoids and anthocyanin content, with 2 mmol L^−1^ MeJA applied 2 or 1 week before harvest (MeJA2, MeJA3) enhancing firmness during cold storage.^105^ For kiwifruit, 0.25 and 1.0 mmol L^−1^ MeJA reduced weight loss, while 0.5 and 1.0 mmol L^−1^ MeJA lowered respiration rates; 1.0 mmol L^−1^ MeJA notably retarded softening and maintained higher vitamin C, total flavonoids and antioxidant activity, although 0.25 mmol L^−1^ MeJA was best for total phenolics.^106^ Overall, higher MeJA concentrations (e.g., 1.0 or 2.0 mmol L^−1^) frequently demonstrated more pronounced beneficial effects on postharvest fruit quality.

### Principal component analysis reveals distinct postharvest responses of ‘Lamuyo’ green peppers to MeJA treatment

**Figure 6:**
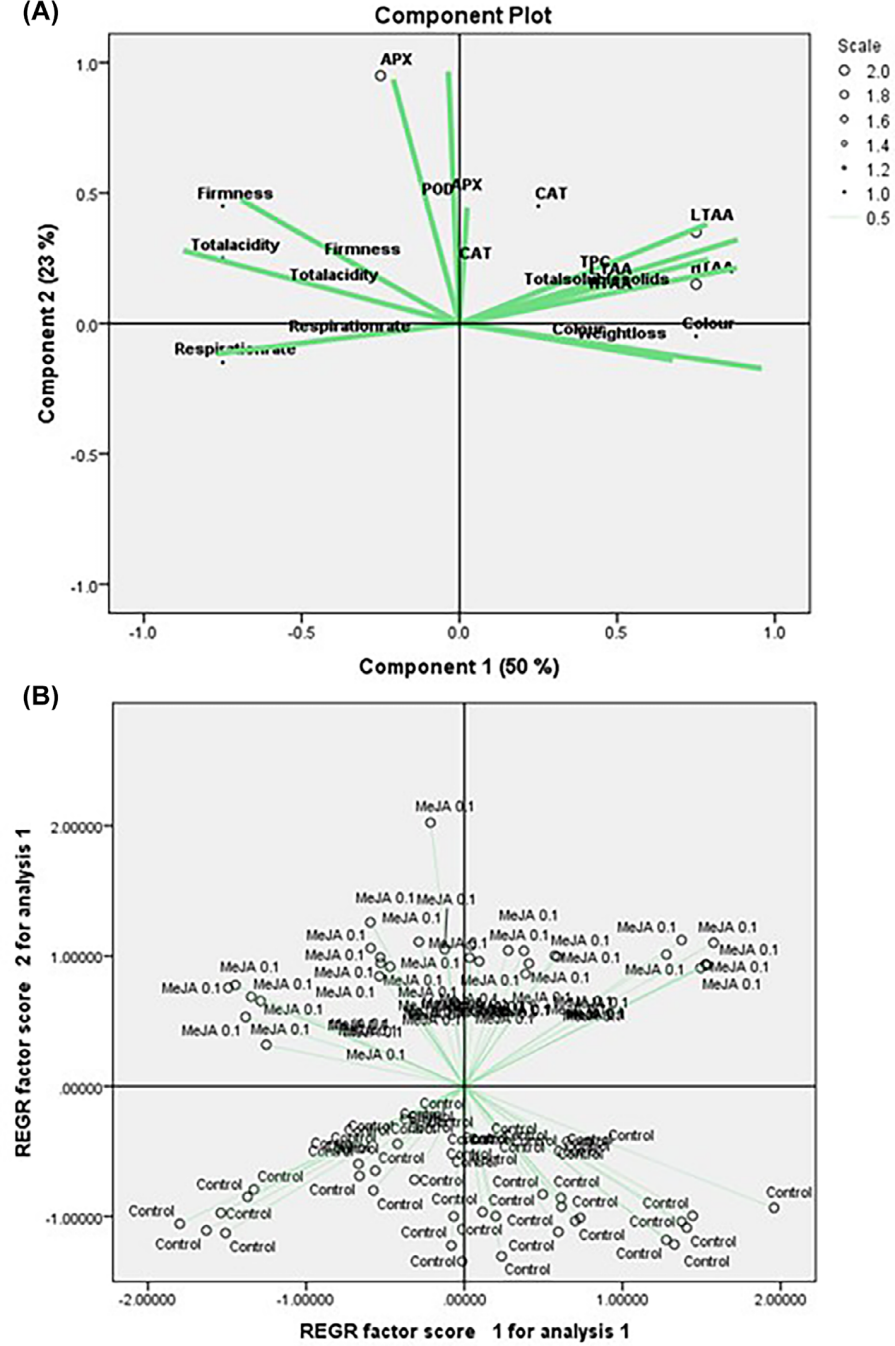
Principal component analysis (PCA) biplot showing the relationships among different parameters measured at harvest and during 28 days of storage at 7 °C (A) and treatments tested (control and 0.1 mmol L^−1^MeJA) (B) in the 2021 season. The two principal components of the PCA explained 73% of the variation in the measured data. The variables measured are abbreviated as follows: TPC, total phenolic content; H‐TAA, hydrophilic–total antioxidant activity; L‐TAA, lipophilic–total antioxidant activity; APX, ascorbate peroxidase; CAT, catalase; POD, peroxidase. Treatments are abbreviated as follows: control and methyl jasmonate (MeJA) at 0.1 mmol L^−1^.

Figure 6(A) shows a component plot derived from principal component analysis (PCA) of various physiological and biochemical parameters in ‘Lamuyo’ green peppers at harvest and during 28 days of postharvest storage at 7 °C. Component 1, which accounts for 50% of the total variance, primarily separates variables related to fruit ripening and senescence. Parameters such as colour, weight loss, H‐TAA and total soluble solids show strong positive loadings on component 1, indicating their association with advancing maturity and deterioration. Conversely, firmness, TA, RR and certain antioxidant enzymes such as APX, CAT and POD exhibit negative loadings, suggesting their link to fruit freshness and antioxidant capacity. Component 2 explains an additional 23% of the variance and highlights other distinctions. APX and CAT show positive loadings, which may indicate their involvement in metabolic pathways not fully captured by Component 1. The proximity of vectors such as APX, CAT and POD indicates a coordinated response of these antioxidant enzymes.

On the other hand, Fig. 6(B) shows the distribution of individual samples within the PCA space, categorised by treatment: control and MeJA at 0.1 mmol L^−1^. This plot, likely a scores plot from the regression factor analysis, reveals a clear separation between the two treatment groups. The control samples are predominantly clustered in the lower left quadrant, characterised by lower scores on both REGR factor 1 and REGR factor 2. In contrast, the 0.1 mmol L^−1^ MeJA‐treated samples are generally located in the upper right quadrant, indicating higher scores on both factors. This distinct separation suggests that MeJA treatment significantly altered the physiological and biochemical profiles of the ‘Lamuyo’‐type green peppers.

## CONCLUSIONS

The preharvest application of MeJA via foliar spraying, conducted over the course of two growing seasons, resulted in a notable enhancement in crop yield for the ‘Lamuyo’ green pepper fruit. This enhancement was observed in two consecutive growing seasons with a lower concentration of 0.1 mmol L^−1^ MeJA. Moreover, the MeJA treatment mitigated losses of weight, firmness, green colour and acidity during prolonged postharvest storage at an optimal temperature of 7 °C. This was achieved by reducing the RR, increasing the TSS content and stimulating the antioxidant system. These effects may be related to a reduction in the level of oxidative stress in the treated pepper fruits, particularly those treated with 0.1 mmol L^−1^. MeJA foliar spraying may also result in enhanced health benefits following the consumption of green pepper fruit by modulating the enzymatic system and the secondary metabolism at the non‐enzymatic antioxidant level. The results demonstrated that the most efficacious concentration for enhancing yield and quality parameters was 0.1 mmol L^−1^, a finding that was corroborated in the second season of 2021. The effects of MeJA on green pepper fruit may have significant commercial implications, as it has the potential to enhance quality at harvest and maintain it during postharvest storage. This may result in a delay in the onset of quality losses and the oxidative stress associated with fruit senescence, thereby extending the shelf‐life of the fruit after prolonged storage at optimal temperatures. Although this is the first investigation to highlight the importance of preharvest MeJA application in regulating physiochemical quality changes during postharvest storage at 7 °C, further molecular insight is required to fully understand the mechanisms involved. To ensure the broader applicability of these findings, future investigations are imperative to evaluate the effects of preharvest MeJA on other *Capsicum annuum* cultivars and types. As our study on ‘Lamuyo’ green bell pepper demonstrates cultivar‐specific responses to MeJA, comprehensive evaluation across diverse pepper varieties is necessary. This will confirm the reproducibility of observed enhancements in yield and quality, maximizing the commercial potential of preharvest MeJA treatments.

## FUNDING INFORMATION

This research received no external funding.

## CONFLICTS OF INTEREST

The authors declare no conflict of interest.

## AUTHOR CONTRIBUTIONS

Conceptualization: AD‐S, MEG‐P and PJZ. Methodology: AD‐S and MJG. Software: MJG. Investigation: AD‐S. Data curation: MEG‐P. Writing – original draft preparation: AD‐S and MEG‐P. Writing – review and editing: MJG, MEG‐P and PJZ. Visualization: all authors. Supervision: MEG‐P and PJZ. Funding acquisition: PJZ. All authors have read and agreed to the published version of the manuscript.

## Data Availability

The data that support the findings of this study are available from the corresponding author upon reasonable request.
